# Efficient Generation of Pancreatic Progenitor Cells from Induced Pluripotent Stem Cells Derived from a Non-Invasive and Accessible Tissue Source—The Plucked Hair Follicle

**DOI:** 10.3390/cells13121010

**Published:** 2024-06-10

**Authors:** Amatullah Fatehi, Marwa Sadat, Muneera Fayyad, Jean Tang, Duhyun Han, Ian M. Rogers, Drew Taylor

**Affiliations:** 1 Department of Physiology, University of Toronto, Toronto, ON M5S 1A1, Canada; amatullah@acorn.me (A.F.); msadat@lunenfeld.ca (M.S.); 2 Lunenfeld Tanenbaum Research Institute, Mount Sinai Hospital, Toronto, ON M5G 1X5, Canada; jeantang@lunenfeld.ca; 3 Acorn Biolabs Inc., Toronto, ON M5G 2N2, Canada; muneera@acorn.me (M.F.); christopher@acorn.me (D.H.); 4 Department of Obstetrics and Gynaecology, University of Toronto, Toronto, ON M5S 1A1, Canada

**Keywords:** induced pluripotent stem cells, regenerative medicine, autologous, non-invasive, keratinocytes, hair follicles, pancreatic progenitor cells

## Abstract

The advent of induced pluripotent stem cell (iPSC) technology has brought about transformative advancements in regenerative medicine, offering novel avenues for disease modeling, drug testing, and cell-based therapies. Patient-specific iPSC-based treatments hold the promise of mitigating immune rejection risks. However, the intricacies and costs of producing autologous therapies present commercial challenges. The hair follicle is a multi-germ layered versatile cell source that can be harvested at any age. It is a rich source of keratinocytes, fibroblasts, multipotent stromal cells, and the newly defined Hair Follicle-Associated Pluripotent Stem Cells (HAP). It can also be obtained non-invasively and transported via regular mail channels, making it the ideal starting material for an autologous biobank. In this study, cryopreserved hair follicle-derived iPSC lines (HF-iPS) were established through integration-free vectors, encompassing a diverse cohort. These genetically stable lines exhibited robust expression of pluripotency markers, and showcased tri-lineage differentiation potential. The HF-iPSCs effectively differentiated into double-positive cKIT^+^/CXCR4^+^ definitive endoderm cells and NKX6.1^+^/PDX1^+^ pancreatic progenitor cells, affirming their pluripotent attributes. We anticipate that the use of plucked hair follicles as an accessible, non-invasive cell source to obtain patient cells, in conjunction with the use of episomal vectors for reprogramming, will improve the future generation of clinically applicable pancreatic progenitor cells for the treatment of Type I Diabetes.

## 1. Introduction

Adult stem cells are undifferentiated cells that are inherent to various tissues and organs in the body [1]. They are involved in the maintenance and repair of tissues, and play a crucial role in the natural healing process of the body [1]. Nestled within distinct microenvironments known as niches, these cells are poised to respond to activation cues when prompted by changes or damage within the environment [2,3]. Upon activation, these dormant stem cells undergo exponential division, generating a reservoir of raw material. Subsequent differentiation of these cells contributes to the restoration of specific cell types essential for niche functionality [3]. A stem cells’ ability to balance quiescence and proliferative activity is critical for their survival [4].

As we age, these populations of stem cells become exhausted [5]. A confluence of factors, including accumulation of reactive oxygen species (ROS), DNA damage, epigenetic shifts, protein damage aggregation, and compromised mitochondrial activity, culminate in stem cell dysfunction and attrition [5]. This results in the need for cell replacement therapies, whereby externally cultivated stem cells are reintroduced into the body, either invigorating existing populations or effecting a comprehensive replacement [6].

Until the early 2000s, the prospect of stem cells in laboratory settings seemed tethered to ethical dilemmas and constrained by the limited availability of suitable cell sources [1,7]. The field of cell replacement was changed when Shinya Yamanaka and Kazutoshi Takahashi demonstrated that differentiated adult cells, specifically adult fibroblasts, could be reprogrammed into a pluripotent cell [8,9]. This discovery, which earned Yamanaka the Nobel Prize in 2012, heralded a new era in stem cell research, shifting the focus from ethically contentious embryonic stem cells to the prospect of autologous induced pluripotent stem cells (iPSCs) [10]. Autologous iPS cell therapies involve the collection of a patient’s own cells from a select tissue source, reprogramming and differentiating them into a therapeutic cell type [11]. Currently, three single-patient human transplants of autologous-iPSC-derived cells have taken place worldwide, with positive phase I results [11].

Autologous therapies provide safer treatments in comparison to their allogeneic counterparts. Direct comparison of autologous and allogeneic transplantation of iPSC-derived neural cells was conducted in the brains of nonhuman primates [12]. The study found that the autologous transplantation elicited only a minimal immune response in the brain [12]. The allografts, however, caused an acquired immune response with the activation of microglia and the infiltration of leukocytes [12]. A higher number of dopaminergic neurons also survived in the autograft [12]. Therefore, autologous cell therapies may facilitate long-term engraftment without the need for immunosuppression. Another major benefit that autologous iPSCs provide is in the field of personalized or precision medicine. Most medical treatments are designed as a one-size-fits-all approach, which may be successful for some patients, but not for others. Precision medicine considers differences in people’s genes, environments, and lifestyles. The goal is to target the right treatments to the right patients at the right time [13].

Overall, the recent significant progress towards regenerating or replacing lost tissue to age, disease, or injury using autologous, induced pluripotent stem cells has led to a paradigm shift in healthcare from palliative to curative treatment. However, we are yet to make the bench-to-bedside concept a reality. This is in part due to the scale-out issues in manufacturing, as well as a lack of streamlined safety and efficacy guidelines [14].

**Figure 1:**
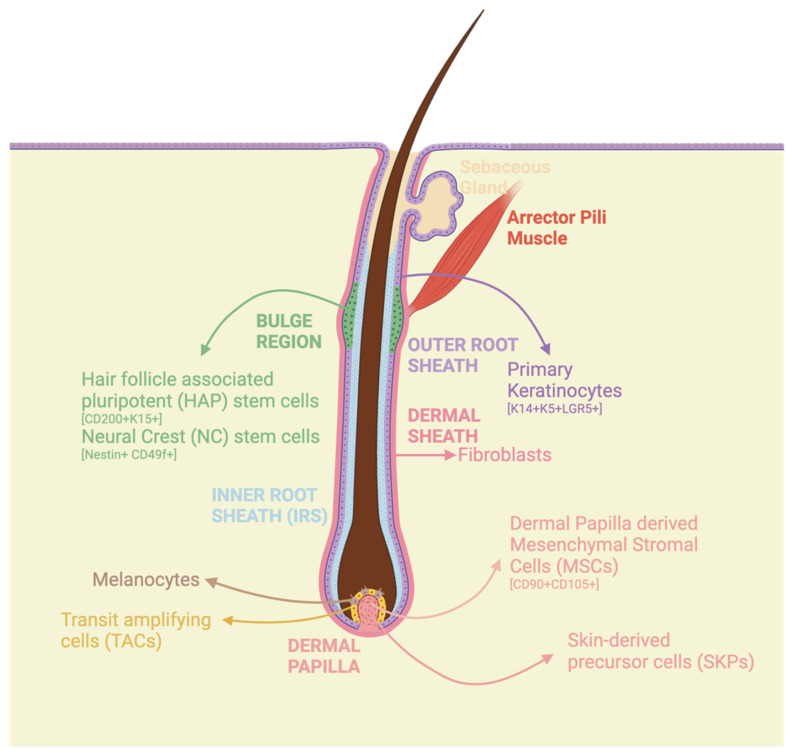
The hair follicle is a unique tissue source with multiple cell populations. The above Figure shows a schematic representation of a hair follicle in the anagen growth phase. The hair follicle contains multiple sub-niches, each encompassing a cluster of multipotent and unipotent cells. The cells include the primary keratinocytes, which make up the outer root sheath (ORS), fibroblasts, mesenchymal stromal cells residing in the dermal papilla, and the newly defined hair follicle-associated stem cells which remain quiescent in the bulge region of the follicle. All the different cells hold immense regenerative medicine potential. The illustration was generated using Biorender.

The hair follicle (HF) is the only multi-germ layered, versatile cell source that can be harvested non-invasively at any age. Termed the “mini organ” due to its complex nature, HFs hold immense regenerative and therapeutic potential [15]. It is a specialized structure located within the skin, housing a complex interplay of different cell types, signaling pathways, and microenvironments. The HF undergoes cyclic patterns of self-triggered regeneration, involving phases of growth (Anagen), regression (Catagen), and quiescence (Telogen) [16]. The anagen phase of the HF cycle harbors key players that orchestrate the regenerative processes. At the bulb of the follicle resides the mesenchymal dermal papilla, while the outer root sheath is populated by primary keratinocytes [17,18,19,20]. The bulge region hosts hair follicle-associated stem cells (HAP) [21,22,23,24]. Surrounding the follicle, dermal fibroblasts and skin-derived precursors (SKPs) contribute to the dynamic microenvironment [17]. This orchestrated ensemble fuels the renewal and morphogenesis of the HF through successive regenerative cycles. (Figure 1).

Over the past decade, the HF has risen to be a notable and versatile tissue source with therapeutic potential. This unique source holds distinct advantages due to its accessibility across various age groups, setting it apart from alternatives like umbilical cord blood, tissue, peripheral blood cells, and deciduous dental pulp [25,26,27,28]. HFs can be collected conveniently through scalp biopsy or plucking, offering a simplified procedure that avoids donor site complications. Patterned hair removal leads to a five times greater regeneration of neighboring hairs through a mechanism known as organ-level quorum sensing, allowing for the collection of ample starting material without donor site morbidity [29]. Chen and colleagues also proposed that pro-inflammatory macrophages are recruited by plucked follicles via CCL2 to regenerate plucked and un-plucked follicles through TNF alpha activation of AKT/β-catenin signaling [29]. The plucking procedure, straightforward and not requiring medical expertise, contrasts favorably with the more structured collection methods essential for bone marrow and adipose tissue.

One possible application is the use of autologous stem cells for the treatment of Type I diabetes (T1D). T1D or insulin-dependent diabetes is a chronic condition where the pancreas makes little or no insulin, as a result of dysfunction of pancreatic, insulin-secreting β-cells [30]. Initially, embryonic stem cell derived, precursor beta cells were combined in an encapsulation device which would help to differentiate the cells towards mature, insulin-producing cells [31]. However, the insulin output was insufficient for clinical benefit, and explants showcased minimal cell survival [32]. The encapsulation device was then modified to allow for the vasculature to grow into its lumen. The implants were well tolerated, and excised grafts showed the differentiation of endoderm cells to mature, functional, β-cell phenotypes [33]. However, patients had to be administered immunosuppressive agents to prevent an allogenic immune response, and insulin independence was not reported in any of the patients [33]. Through this work, scientists were able to understand the genes and signaling pathways responsible for the coordination of events leading to β-cell differentiation and maturation. In 2016, Millman et al. confirmed that β-cells derived from reprogrammed skin fibroblasts functionally resembled adult islet β-cells both in-vivo and in vitro [34]. Since then, the work has been repeated on multiple human stem cell lines [35].

Our study delved into the feasibility and constraints of utilizing plucked hair follicles for generating clinical-grade autologous-induced pluripotent stem cell (iPSC) lines, irrespective of age or sex. In our investigation, we engaged 28 participants encompassing varied ages, whose hair follicles were previously collected and cryogenically stored in a biobank. Employing an explant culture approach, we isolated and expanded the preserved cells. Keratinocytes cultured from the excised hair follicles were then subjected to reprogramming using an integration-free, viral-free vector system. The reprogrammed cells underwent rigorous validation for pluripotency, adhering to established industry benchmarks. To assess their clinical promise, we directed the differentiation of these cells toward the pancreatic lineage.

## 2. Materials and Methods

### 2.1. Ethics Statement

This research was conducted following the approval of the Mount Sinai Hospital Research Ethics Board (22-0090-E: Investigating the production of islets from induced pluripotent stem cells (iPSC) (eSubmission) granted on 19 July 2022. Informed consent was obtained from all participants, who were informed of the study’s objectives, potential risks, and benefits. Written informed consent was obtained from all participants before their participation. The study was carried out in accordance with the recommendations in the Canadian Council on Animal Care Guidelines. Animal protocols were approved by the Mount Sinai Hospital Research Ethics Board (22-0090-E).

### 2.2. Collection and Cryopreservation of the Plucked Hair Follicles

Hair Follicles were obtained from Acorn Biolabs, Inc. Briefly, Acorn Biolabs collected hair follicles from the occipital lobe of 28 participants of various ages and sex (see Supplementary Table S1). The participants consented to the collection of their follicles, and the subsequent use thereof, in accordance with the guidelines of the Mount Sinai Hospital Research Ethics Board. The follicles were collected using a plucking motion, transported in Acorn’s Cell Transportation MediumTM (Gibco, Emeryville, CA, USA), and cryogenically frozen in an ISO-7 processing suite according to the International Organization for Standardization (ISO) 14644-1:2015 [36]. Viability scores were obtained for each sample prior to freezing using the ImageXpress Micro Confocal System. The follicles were frozen in liquid nitrogen under vapor phase (−194 °C).

### 2.3. Live–Dead Viability

In total, 3–5 follicles were taken for viability analysis. Follicles were placed in 100 μL of Live Dead Cytotoxicity solution (Invitrogen, Carlsbad, CA, USA) for 15 min in the dark. The solution was decanted, and 100 μL of Hoechst33342 solution (Invitrogen, USA) was added. The follicles were incubated in the dark for an additional 5 min. The follicles were washed in 1 mL of DPBS without Mg^2+^ and Ca^2+^ (Gibco, USA). The follicles were cut down to the ORS and placed in black 96-half well plates (Nunc, Rochester, NY, USA) containing 10 μL of DPBS without Mg^2+^ and Ca^2+^. Special care was taken to ensure that the follicles did not float. The plate was analyzed using the ImageXpress Confocal microscope system. Parameters were pre-set to capture cells positive for the FITC (live cells), Texas Red (dead cells) and DAPI (total cells) wavelengths. Total cell counts (DAPI^+^) and live cell counts (FITC^+^) for 22 participants were assessed against viability scores obtained prior to cryopreservation with the PRISM software (version 9.3.1) (paired *t*-test; *p*-value; two-tailed).

### 2.4. Primary Cell Culture Using the Explant Culture Method

Participant samples were removed from liquid nitrogen, and were quickly thawed in a 37 °C water bath for 2–3 min each. The samples were transferred into 35 mm sterile Petri dishes (Thermofisher, Waltham, MA, USA) containing CTS-DPBS without Mg^2+^ and Ca^2+^ (Gibco, USA), and then were washed for 3 min in the solution. The dishes were transferred into the ISO-7 cleanroom for further processing. For each participant, 10 follicles were cut and placed in the middle of a 48-well tissue culture plate (Nunc, Roskilde, Denmark) coated with Matrigel (Corning, New York, NY, USA). Only follicles containing an intact ORS were selected. To mitigate against the follicles drying, a drop of KSR (20% media knockout serum replacement in DMEM/F12, 1× MEM Non-Essential Amino Acid, 0.1 mM β-Mercaptoethanol, 1× Antibiotic Antimycotic) was added to each well prior to plating. Once all the follicles were plated, the media were removed, and a fresh drop of media (50 μL) containing 10 ng/μL bFGF (Thermofisher, USA) was added on top of each follicle. The plates were placed in a 37 °C incubator at 5% CO_2_ overnight. In total, 3–5 follicles from each participant were used for the viability analysis. The next morning, each well was flooded with 150 μL of media. A complete media change was conducted every other day for 14 days. The plates were observed using a phase contrast microscope for potential outgrowths every day. Outgrowths, arising from the bulge of the follicle, were observed from day 4 onwards. Any outgrowth observed was recorded. The percentage of outgrowth (# of follicles with positive outgrowth/total # of follicles plated) was calculated for each participant.

### 2.5. Expansion of Keratinocytes Using the Feeder-Free Method

At 75% confluency, cells were enzymatically dissociated using 200 μL of TrypLE Express (Gibco, USA) per well. The suspension of cells was pooled for each participant, and was centrifuged at 300× *g* for 5 min. The resulting supernatant was discarded, and the pellets were resuspended in 1 mL of DermaCult Keratinocyte Expansion Medium supplemented with Hydrocortisone (Stemcell Technologies, Vancouver, BC, Canada) each. The solution was added to a 12-well tissue culture plate (Greiner bio-one, Germany) coated with Rat Tail Collagen (Gibco, USA). The cells were incubated in a 37 °C incubator at 5% CO_2_, and the media were changed every other day. The cells were expanded until 80% confluency, and then were passaged onto 6-well tissue culture plates at a confluence of 3 × 10^4^/cm^2^ (Greiner bio-one, Frickenhausen, Germany). At each passage, cells were counted using a hemocytometer, and keratinocyte expansion rates were extrapolated. On passage 3, the cells were taken for reprogramming.

### 2.6. Reprogramming of Keratinocytes Using the Non-Viral Reprogramming Method

Keratinocytes between passage 2 and 3 were taken to the Lunenfeld Tanenbaum Research Institute for reprogramming from multiple participants (***N* = 11**). Then, 24 h prior to reprogramming, cells were incubated in DermaCult media supplemented with Hydrocortisone without antibiotics and antimycotics. The cells were enzymatically dissociated using TrypLE Express (Gibco, USA) at 37 °C for 5 min. The dissociation was stopped with equal amounts of EpiLife CF (Gibco, USA) supplemented with EDGF and 0.3 μM Ca^2+^. The cell solutions were centrifuged at 300× *g* for 5 min. Viable cells were counted using a hemocytometer and 5 × 10^5^ cells/well were taken per reprogramming event. The cells were pelleted and resuspended in 100 μL of Resuspension Buffer R (Invitrogen, USA) on ice. In total, 1 μL of Epi5 Reprogramming Vectors (Oct4, Sox2, Klf4, L-Myc, and Lin28) and 1 μL of Epi5 p53 and EBNA vectors (Life Technologies, Carlsbad, CA, USA) were added to each 100 μL solution. The solution was electroporated using the NeonTm Transfection System (Thermofisher, USA) at a pulse voltage of 1150 for 30 milliseconds and 2 pulses. The solution was then directly seeded onto 6-well tissue-treated (Falcon, Chicago, IL, USA) Matrigel (Corning, USA)-coated plates containing 2 mL of EpiLife CF media supplemented with EDGF and 0.3 μM Ca^2+^. The plates were incubated in a 37 °C incubator at 5% CO_2_. After 24 h, the media were changed to N2B27 media (DMEM/F12, 100X N2, 50X B27, 10 mM MEM NEAA, 100X Glutamax, 0.1 mM β-Mercaptoethanol, 10 ng/mL bFGF) and were supplemented with 10 ng/μL bFGF sequentially over the course of 7 days (i.e., on Day 1, 1 mL of N2B27 media were added without removing any media; on Day 2–4, 50% of the media were removed, and fresh media were added, with a media change every other day; and on Day 4–6, 75% of the media were removed, and fresh media were added, with a media change every other day). On Day 7, the media were changed completely to N2B27 media. On Day 9, the media were changed to TeSR1 media (Stem Cell Technologies, Vancouver, BC, Canada). The media were changed every other day for the next 12 days. The plates were also observed under a phase contrast microscope for colony formation.

### 2.7. Selection and Expansion of iPS Cells

On Day 21, the number of colonies present in each participant’s well was identified using phase contrast microscopy. An equivalent number of wells were coated using vitronectin (Gibco, USA). Colonies were picked using a 200 μL pipette tip, and then were placed directly in a well containing mTeSR Plus media (Stem Cell Technologies, Catalog #100-0274, Canada) supplemented with 1× Antibiotic Antimycotic (100×) solution (Gibco, USA) and 10 μM Rock_i_. The plates were incubated in a 37 °C incubator at 5% CO_2_, and the media were changed every other day with fresh mTeSR Plus media without Rock_i_. The clones were grown for one week, and wells containing cells were treated with ReLeSR (Stem Cell Technologies, Cambridge, MA, USA) for 1 min at room temperature. The solution was aspirated, and the plates were kept at 37 °C for 3 min. The dissociation was stopped using fresh mTeSR Plus media supplemented with 10 μM Rock_i_. The solution was added directly to a 24-well plate coated with vitronectin. The cells were grown to 70–80% confluency (Passage 2). Individual clones for every participant were dissociated using ReLeSR, and then were cryogenically frozen with CryoStor media containing 10% DMSO (Stem Cell Technologies, USA) using the slow-rate cooling method. A select number of participants’ (*N* = 4) colonies were taken further for expansion. From passage 2 onwards, clones were expanded using a 1:10 split ratio. Approximately 1 × 10^5^ cells were plated into 1 well of a 6-well plate (VWR, Radnor, PA, USA) (~1.05 × 10^4^ cells/cm^2^) and then were cultured for 4–5 days in a 37 °C incubator at 5% CO_2_ for 10 passages.

### 2.8. Episomal Vector Clearance Detection Using PCR

DNA was isolated from iPSCs by lysing cells in 100 μL of 0.05 M NaOH and incubating them for 10 min at 98 °C. The reaction was then neutralized using 10 μL of 1 M Tris (pH 8.0), and the DNA was further diluted by adding 100 μL of molecular-grade sterile water (Wisent, Quebec, QC, Canada). DNA concentrations were measured using Nanodrop. Custom primers were purchased from Eurofin. OriP and EBNA-1 were used for the detection of the Epi5 vectors, and GAPDH was used as a house-keeping gene. PCR samples were then run on a 1.5% agarose gel (FroggaBio, Concord, ON, Canada) with 1× Sybrsafe dye (Invitrogen) and 1000 bp ladder (MBI Fermentas, Glen Burnie, MD, USA). All of the gels were visualized using UV fluorescence.

### 2.9. Trilineage Differentiation of iPS Cell Lines

Cells from the 4 cell lines were seeded for Endoderm, Mesoderm, and Ectoderm differentiation using the STEMdiff Trilineage Differentiation Kit (Stem Cell Technologies, Canada) on 24-well black ibidi plates coated with ready-to-use Geltrex solution (Gibco, USA). Each cell line was grown to 70–75% confluency, the media were removed, and the cells were washed with DPBS without Mg^2+^ and Ca^2+^. The cells were then trypsinized using 1 mL of Gentle Cell Dissociation Reagent (Stem Cell Technologies, Canada) for 8 min at 37 °C. The cells were transferred to a tube containing 1 mL of DMEM/F-12 (Stem Cell Technologies, Canada) and were centrifuged at 300× *g* for 5 min. The supernatant was removed, and the cells were resuspended in Single-Cell Plating Medium (mTeSR 1 supplemented with 10 μM Rock_i_). The viable cells were counted using the Countess 3 (Thermofisher). In total, 200,000 cells/cm^2^ cells from each cell line were seeded in 3 wells for both Ectoderm and Endoderm differentiation. An amount of 50,000 cells/cm^2^ were seeded in 3 wells for Mesoderm differentiation. For Endoderm and Mesoderm differentiation, the media was changed daily for 4 days. The cells were fixed on day 5. For Ectoderm differentiation, the media were changed daily for 6 days. On day 7, the cells were washed, fixed, and characterized using immunofluorescence.

### 2.10. Teratoma Xenograft Assay

Cells from all 4 lines were grown in 6-well plates (X4). At 75–80% confluency, the cells were harvested, counted, and mixed 1:1 with COLD Matrigel (Corning, USA) on ice. In total, 1 × 10^7^ cells were injected subcutaneously into the dorsal flank of each NOD/SCID Gamma mice. An amount of 3 mice were injected per cell line. The mice were monitored for tumor growth over the course of 8–12 weeks. All mouse injections, as well as the monitoring of tumors, were carried out by Jean Tang in accordance with the guidelines of the Animal Care Committee. The tumors were excised and processed at The Center for Phenogenomics (TCP). Tissue structures were analyzed after Hematoxylin and Eosin (H&E) staining by the Pathologist Dr. Mohamed Eskandarian at TCP.

### 2.11. Assessment of Genetic Stability through Karyotyping

All four of the hair follicle iPSCs lines were sent to the Cytogenomic Services at the Hospital for Sick Children for karyotyping. Karyotype analysis via G-banding was performed on cells from 3 wells of a 6-well plate per cell line. The preparation of cells was carried out by Raymond Wong, according to the parameters below. Routine G-banding analysis was carried out, and 15 metaphases per cell line were examined. The results were analyzed and interpreted by Raymond Wang.

### 2.12. Direct Differentiation towards Pancreatic Lineage

HF-iPS cell lines were differentiated to definitive endoderm cells using the STEMdiff pancreatic progenitor kit (Stem Cell Technologies, Canada) along with a control Human ES cell line (CA-1). iPS cell lines were grown to 70–75% confluency on 6-well tissue culture plates coated with Cultrex (R&D Systems, Minneapolis, MN, USA). The cells’ wells were washed once with DPBS without Mg^2+^ and Ca^2+^. The washing medium was replaced with Medium 1A (Endoderm Basal Medium + Supplement MR + Supplement CJ). On day 2, the cells were switched to Medium 1B (Endoderm Basal Medium + Supplement CJ). Fresh media was added for 4 days. On day 6, the cells were washed with DPBS without Mg^2+^ and Ca^2+^, and the cells were taken for immunohistochemistry and FLOW cytometry analysis. For a single line, cells were carried further towards pancreatic progenitor cells. On day 5, the media was switched to Stage 2–4 Endoderm Media supplemented with Supplement 2A and 2B (Stem Cell Technologies, Canada). The following day, the media was replaced, and was only supplemented with 2B. On day 8, the media were switched to Stage 2–4 Endoderm Media supplemented with Supplement 3. From days 9–13, the media was changed each day, and was replaced with Stage 2–4 Endoderm Media supplemented with Supplement 4. On day 14, the differentiation was stopped, and the purity of the cells was assessed using immunohistochemistry.

### 2.13. Flow Cytometry

The DE cells were trypsinized using Accutase (Stem Cell Technologies, USA). The cells were centrifuged at 300× *g* for 5 min, and the pellet was suspended in a cell-staining buffer (2% FBS in PBS) and pre-incubated with 5 μL of Human TruStain FcXTm (BioLegend, San Diego, CA, USA) for 10 min at room temperature. Conjugated fluorescent probes (see Supplementary Table S2) were added to each reaction and incubated on ice for 20 min in the dark. The cells were then washed twice and centrifuged at 350× *g* for 5 min. The cells were then incubated with 0.4 μg/mL DAPI (Sigma, St. Louis, USA) for 5 min in the dark. FLOW cytometry was performed by Annie Bang at the Toronto Center for Phenogenomics (TCP) using the Gallios Flow Cytometer system (Beckman, Indianapolis, USA).

### 2.14. Immunofluorescence

Immunofluorescent assays: Cells were fixed with 3.7% PFA (Sigma Aldrich, St. Louis, MO, USA) for 20 min at room temperature. The cells were washed once with DPBS without Mg^2+^ and Ca^2+^, and then were permeabilized with Permeabilization Buffer (0.1% TritonX-100) for 10 min at room temperature. The cells were washed 3X with a washing buffer (DPBS without Mg^2+^ and Ca^2+^) for 5 min each. Once permeabilized, the cells were incubated with Blocking Buffer (1% BSA in DPBS without Mg^2+^ and Ca^2+^) for 1 h on a rocker. The cells were washed once again with a Washing Buffer (3×) for 5 min each. The cells were incubated overnight with primary antibodies (see Supplementary Table S3) at 4 °C in a primary antibody solution. After 24 h, the primary antibody solution was removed, and the cells were washed with Washing Buffer 3X for 5 min each. The cells were incubated with secondary antibodies (see Supplementary Table S3) on a rocker for 1 h in the dark. After secondary incubation, the cells were washed with Washing Buffer 3× for 5 min each. The cells were incubated with Hoechst 33342 Solution for 5 min in the dark. The solution was removed and replaced with DPBS without Mg^2+^ and Ca^2+^. The plates were imaged using the IXM Confocal System (Molecular Devices, San Jose, CA, USA) using the DAPI (377/54 nm), FITC (475/34 nm), and Cy5 (631/28 nm) wavelengths.

### 2.15. Statistical Analysis

Participant data were analyzed using the PRISM software (version 9.3.1). The percentage of outgrowth rates, quantified using the number of follicles with outgrowths divided by the total number of follicles plated for each participant, was matched against participant demographics (i.e., sex and age). To begin with, percent outgrowth was plotted against participant age using a Pearson r correlation analysis. Participants were then divided into male [*N* = 14] and female [*N* = 14] according to the participants’ identification of biological sex. The percentage of outgrowth was analyzed against sex using an unpaired *t*-test (*p*-value < 0.05). Participants were further divided into two groups according to age. Participants between the ages of 18 and 40 were arbitrarily placed in one group designated to be the “younger” age group [*N* = 17], and participants older to 40 years of age [*N* = 11] were placed in a second group, designated to be the “older” age group. The groups were re-analyzed using an unpaired t-test (*p*-value < 0.05). Live cell and total cell counts pre- and post-cryopreservation [*N* = 20] were also analyzed against the percentage of outgrowth rates using unpaired *t*-tests (*p*-value < 0.05). For the comparison of reprogramming rates, the number of colonies formed and persisted to P2 were identified for each of the 11 participants. The number of colonies were examined against a couple of parameters using the PRISM software (version 9.3.1). Colony formation, defined as the number of colonies observed after 21 days of reprogramming, was correlated to age [*N* = 11], total cell count pre-cryopreservation [*N* = 9], live cell count pre-cryopreservation [*N* = 9], and the percentage of outgrowth [*N* = 11] using a linear regression model. The number of colonies formed by each sex was also compared using an unpaired *t*-test [*N* = 11].

## 3. Results

### 3.1. Primary Keratinocytes Can Be Isolated from Cryopreserved Plucked Hair Follicles Using a Serum-Free, Feeder-Free, Explant Culture Method

**Figure 2:**
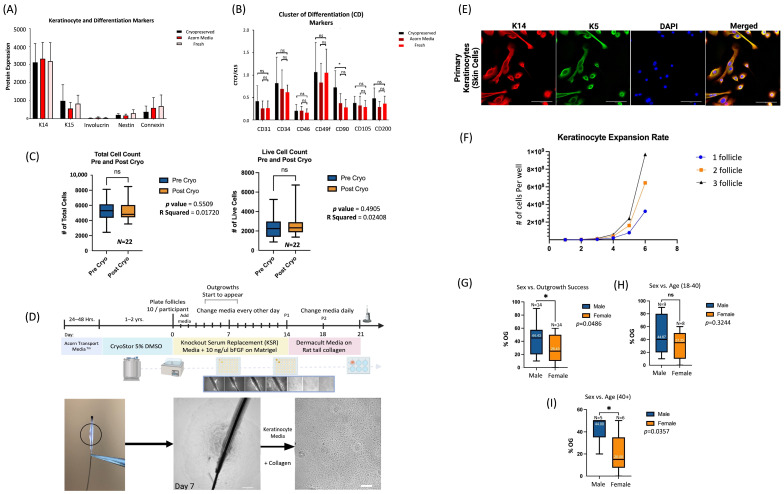
Primary keratinocyte expansion from cryopreserved plucked follicles. Whole, plucked follicles from several of participants [*N* = 5] were placed in Acorn Transport Media Tm for 24–48 h, and were then cryogenically frozen. The follicles were analyzed for protein expression using immunofluorescence after collection, transport, and cryopreservation. The follicles showed no significant change in keratinocyte marker expression (K14, K15) across the process of collection, transport, and cryopreservation (**A**). The follicles also showed no significant (ns) change in CD marker expression reflective of mesenchymal (CD90, CD105) and neuroepithelial cell populations (CD49f) relative to K15 (**B**). Additionally, follicles from 28 participants which had previously been transported in Acorn Transport Media^TM^ and cryogenically frozen were thawed. A subset of five follicles from 22 participants were assessed for viability. There was no significant (ns) difference in total and live cell counts pre- and post-cryo-preservation (**C**). Ten follicles from each participant were then cultured using a modified explant culture method over the course of 21 days. Scale bars = 200 µm (**D**). Intact anagen hair follicles with a visible bulge were plated on Matrigel-coated plates. Outgrowths were observed, originating from the bugle on average, around day 7. The primary cells were then sub-cultured on collagen-coated plates around day 14. The selected keratinocytes were then further passed for 2–3 passages. The cells were positive for K14 and K5 marker expression when assessed through immunofluorescence, indicative of primary keratinocytes. Scale bars = 100 μm (**E**). The primary keratinocytes were expanded up until passage 6, and cell counts were assessed. One outgrowth resulted in 1.2 million cells by passage 2, a quantity sufficient for reprogramming (**F**). Percent outgrowths (# of follicles with positive outgrowth/10) were evaluated across both males and females (**G**). There was no significant (ns) difference when looking at the younger demographic (<40) (**H**). However, there was a significant (*) difference in the older demographic (>40), with females having, on average, a lower percentage of outgrowth (**I**).

Prior to the start of the study, it was important to understand the effect of collection, transport, and cryopreservation on hair follicles. To rule out any adverse effects on the follicles because of the banking process, a sample of plucked hair follicles were collected and analyzed using immunofluorescence using confocal microscopy. The follicles showed no significant difference in protein marker expression after transport and cryopreservation (Figure 2A,B). The plucked follicles showed high expression of cytokeratin 14, 15, and CD49f, indicating toward the retention of the outer root sheath and a mesenchymal stromal cell population, respectively. Cryopreserved hair follicles from 28 participants were thawed, and samples for each were analyzed for viability. There was no significant difference in total and live cell count after cryopreservation (Figure 2C). An average of 3000 live cells were retained across multiple participants (Figure 2C). Overall, the cryopreservation process does not significantly affect the immunophenotype of the follicle.

Cells from the follicle were isolated and expanded using a modified explant method from Aasen et al. [37]. Modifications from the initial protocol (Figure 2D) helped to prevent premature differentiation of keratinocytes in a calcium-rich medium, and allowed for the retention of a heterogeneous population of cells that could aid in cell signaling and proliferation. The primary cells were seen originating from the bulge around day 4–7 (Figure 2D). The cells were then expanded on collagen-coated plates in serum and BPE-free media (Figure 2D). The expansion culture system allowed for the selection and expansion of primary keratinocytes, as seen by the co-labelling of basal markers K14 and K5 (Figure 2E). This persisted up until passage 8, after which the keratinocytes developed a flattened morphology and started to experience signs of terminal differentiation and senescence, including the loss of K14 and K5 expression. A singular follicular outgrowth for certain participants led to 1.2 million cells by passage 2 (Figure 2F). Follicles were plated using cGMP alternatives for a subset of participants. Matrigel was substituted for equivalent amounts of CELLstart substrate, which is a xenofree alternative. The follicles resulted in outgrowths for all the plated individuals, which indicates the feasibility of a clinical-grade protocol for cellular expansion from cryopreserved hair follicles. Our findings show that cryopreserved follicles are adequate to generate the number of primary keratinocytes required for reprogramming.

### 3.2. More Cellular Outgrowth Is Observed from Hair Follicles Obtained from Younger Males

The participants were split into two cohorts according to sex (male and female). The percent outgrowths for both cohorts were analyzed using an unpaired *t*-test. There was a significant difference between males and females. Females have less outgrowth success than males. Females have an average of 20% outgrowth success, while males have an average of 40% outgrowth success (Figure 2G). The sex difference was more evident when both groups were further sorted into young (18–40) and old (40+). A difference was only observed in females in the older age group (40+) (Figure 2G).

### 3.3. Plucked Hair Follicle-Derived Keratinocytes Can Be Easily Reprogrammed Using Episomal iPSC Reprogramming Vectors

**Figure 3:**
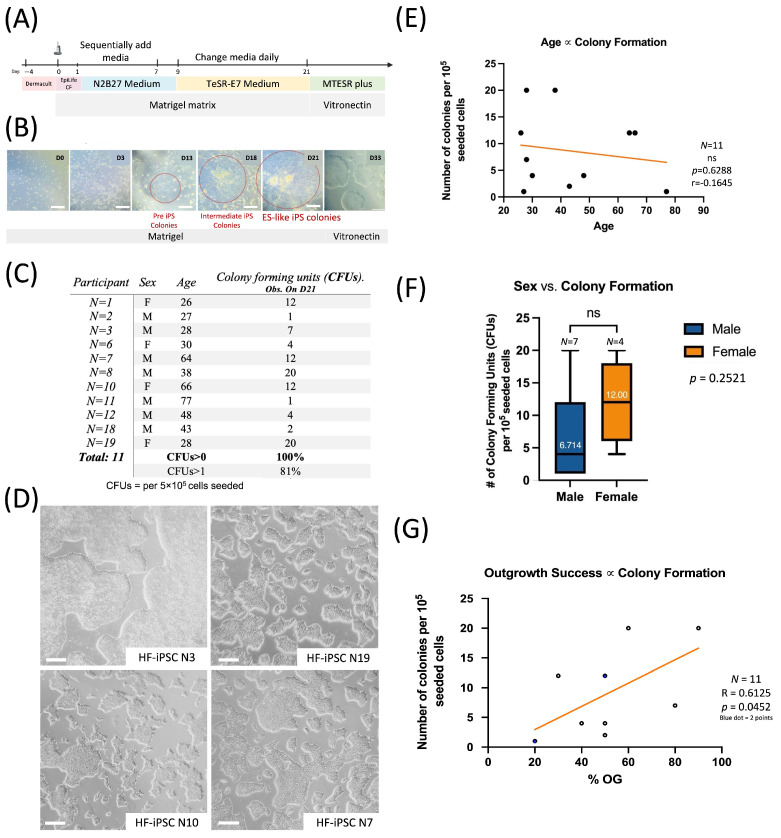
Cryopreserved hair follicle derived primary keratinocytes can be reprogrammed using a feeder-free system across multiple demographics. Expanded primary keratinocytes (5 × 10^5^ cells) from 11 participants were taken for reprogramming at passages 2–3 (**A**). The cells were electroporated and reprogrammed using the Epi5 vector system and gradually placed into N2B27 media. At day 9, the media were switched to TeSR E7, after which the development of Embryonic Stem Cell (ESC)-like colonies was observed until day 21. The non-reprogrammed keratinocytes flattened and ceased to proliferate in culture. Scale bar = 100 µm and 200 µm for d33 (**B**). All 11 participants were able to generate at least one colony, which could be further expanded by day 21 (**C**). In total, 4 participant cell lines from the older (>60) and younger demographic (<40) were further expanded, and episome-free lines were generated. All the cell lines exhibited ESC morphology. Scale bar = (**D**). The reprogrammed participants’ ages were correlated to their reprogramming efficiency (# of colonies formed on day 21). There was no significant difference observed (**E**). There was also no significant difference between reprogramming efficiency and sex, with both sexes having similar colony-forming units. (ns: non-significant.) (**F**). There was a significant positive correlation when comparing reprogramming efficiency and % outgrowth success (# of follicles with outgrowth/10) (**G**).

Of the 28 participants, 11 participants were chosen at random, and the expanded keratinocytes were taken from passages 1 to 3 toward reprogramming. A protocol was developed to reprogram hair follicle-derived keratinocytes, based off the protocol used to reprogram umbilical cord tissue (Figure 3A) [25]. Pre iPS colonies began emerging around day 13. By day 21, the colonies were distinct in size and morphology (Figure 3B). Partially reprogrammed keratinocytes, as well as those which had not reprogrammed successfully, began to terminally differentiate. Terminal differentiation of keratinocytes occurs due to high calcium concentrations, like that seen in the N2B27 transition media. Terminal differentiation leads to a flattened morphology, which is very distinct from the rounded morphology seen for embryonic-like stem cells or iPSCs. Terminal differentiation also leads to senescence, and keratinocytes cease to proliferate. This creates an optimal situation for the manual transferring of colonies and their subsequent iPS passaging. Due to the stark difference in morphology, emerging colonies are easily identified. The colonies were compact, with distinct borders and well-defined edges (Figure 3D). The cells also had a large nucleus-to-cytoplasm ratio, which was observed using phase contrast microscopy. This morphology is consistent with those observed of human embryonic stem cells (hESCs). From this point forward, the cells could be expanded using ReLeSR, allowing for easy serial passaging of optimally sized aggregates. In total, 100% of the participants were able to form at least one colony by day 21 (Figure 3C).

### 3.4. Patient Specific iPS Cell Lines Can Be Generated from Any Adult-Aged Individual Using Cryopreserved Hair Follicles

In the literature, it was found that donor age may reduce reprogramming efficiency, but not impact the ability of the cells to differentiate. Each cell source comes with an inherent set of donor mutations which persist through iPS generation and maintenance [38,39]. In addition, stochastic mutations during the culturing process increase linearly, and older donors do not have the sufficient repair mechanisms to combat these mutations [40]. However, it was established that there was no significant correlation between donor age and colony formation when reprogramming hair follicle cells using the episomal vector system (Figure 3E). There was also no significant correlation between donor sex colony formation (Figure 3F). However, there was a negative trend observed when comparing age and colony-forming units (Figure 3E). This means that an iPS cell line can be produced for any adult aged person (18–65 years of age), with older individuals having fewer colony-forming units per reprogramming reaction (Figure 3E). To further evaluate this, more donors from each age group would need to be taken for reprogramming. One significant finding is that there is a significant positive correlation between outgrowth success and reprogramming efficiency (Figure 3G). This indicates that the quality of starting material matters, and that certain cells may be primed for reprogramming. Overall, our feeder-free, non-integrated reprogramming method is highly advantageous, and works across a diverse group of participants. It is inexpensive, requires little technical effort, and is applicable in pre-clinical research.

### 3.5. Hair Follicle-Derived iPSCs Are Cleared of Episomal Vectors after Serial Passaging, While Retaining Pluripotency Marker Expression and Genetic Stability

**Figure 4:**
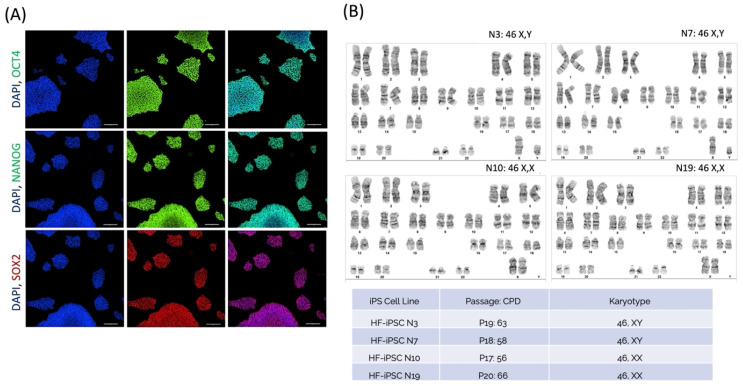
Induced pluripotent stem cells derived from cryopreserved hair follicles maintain pluripotency in vitro and are genetically stable. The episome-free lines were evaluated for pluripotency marker expression using immunofluorescence and confocal imaging. All the cell lines were positive for OCT4 (Green = FITC488]), Nanog (Green = FITC488), and SOX2 (Red = CY5647), as represented by cell line N3. Scale Bars = 200 μm (**A**). The cells were then expanded at various passages and cell population doublings (CPD), and were analyzed using G-banding analysis. Routine G-banding analysis was carried out, and 15 metaphases per cell line were examined. All four cell lines had no major chromosomal abnormalities, and the lines exhibited their respective XY: XX chromosome patterning (**B**).

After establishing the validity of the reprogramming method, four participant cell lines were expanded and further characterized. Two participants over the age of 60 years and two participants under the age of 40 years were randomly selected. Each independent clone was expanded, and cells from passage 5 onward were analyzed for Episomal vector clearance using polymerase chain reaction (PCR) analysis. Some cell lines were episome-free as early as passage 7, while others were episome-free around passage 15. The lines expressed high levels of OCT4, NANOG, and SOX2 (Figure 4A). To assess the genomic stability of the cryopreserved HF-iPSCs, karyotype analysis was performed on all the cell lines. The lines were grown to 15–19 passages or 55–70 population doublings, and were sent to SickKids Hospital, where 20 metaphase cells were examined using G-band analysis from cytogenetic preparations to verify any gross abnormalities. Normal karyotypes were observed for the cell lines, regardless of donor age (Figure 4B). The major abnormalities observed in reprogrammed cell lines, such as Trisomy 12, were not observed.

### 3.6. Hair Follicle Derived iPSCs Have Trilineage Potential In Vitro and In Vivo, Allowing for the Generation of Multiple Cell Types

**Figure 5:**
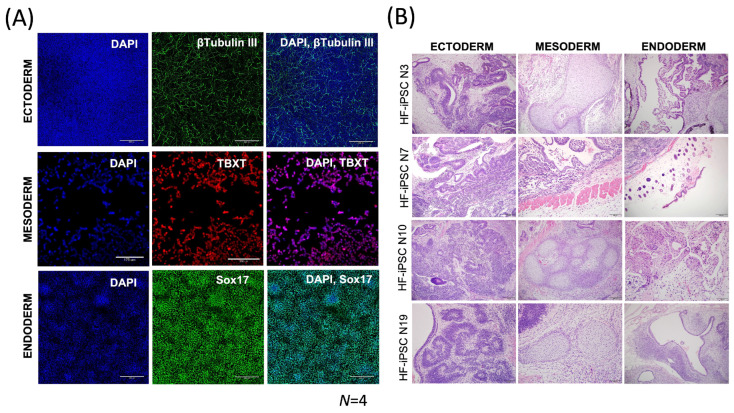
Induced pluripotent stem cells derived from cryopreserved hair follicles show tri-lineage potential both in vitro and in vivo. Cell lines were expanded and directly differentiated towards the Ectoderm, Mesoderm, and Endoderm lineages for 6, 3, and 4 days, respectively. Immunofluorescent staining was used to analyze the expression of TUJ3 (Ectoderm; Green = FITC488; Scale bars = 300 μm), TBXT (Mesoderm; Red = CY5647; Scale bars = 175 μm), and SOX17 (Endoderm; Green = FITC488; Scale bars = 300 μm) for all lines (**A**). The cell lines were simultaneously expanded and subcutaneously injected into NOD SCID gamma mice. Tumor growth was observed over the course of 8–12 weeks, and palpable tumors were excised, sliced, and analyzed using Hematoxylin and Eosin (H&E) staining. H&E staining confirmed the presence of neuroepithelial (Ectoderm)-, gut epithelial (Endoderm)-, and cartilage (Mesoderm)-like structures for all four cell lines. Scale bars = 200 μm (**B**).

In this study, cryopreserved HF-iPSCs were differentiated into the three germ layers (ectoderm, endoderm, and mesoderm) using a commercially available kit to assess their differentiation capacity. Immunofluorescent staining was used to analyze the expression of TUJ3 (Ectoderm), Brachyury (Mesoderm), and SOX17 (Endoderm) markers (Figure 5A). The cells were cultured in specific media for 4 days (endoderm), 3–4 days (mesoderm), and 6 days (ectoderm), with positive expression observed for all three markers in all cell lines. To assess the pluripotency of the cell lines, roughly 1 × 10^7^ cells per line were expanded, mixed with Matrigel, and subcutaneously injected into the flanks of NOD/SCID Gamma mice for the teratoma xenograft assay. The tumors developed over the course of 8–12 weeks, after which they were excised, fixed, and stained. H&E staining confirmed the presence of neuroepithelial (Ectoderm)-, gut epithelial (Endoderm)-, and cartilage (Mesoderm)-like structures for all four cell lines (Figure 5B).

### 3.7. Hair Follicle Derived iPSCs Can Be Differentiated towards Pancreatic Lineages with High Efficiency

**Figure 6:**
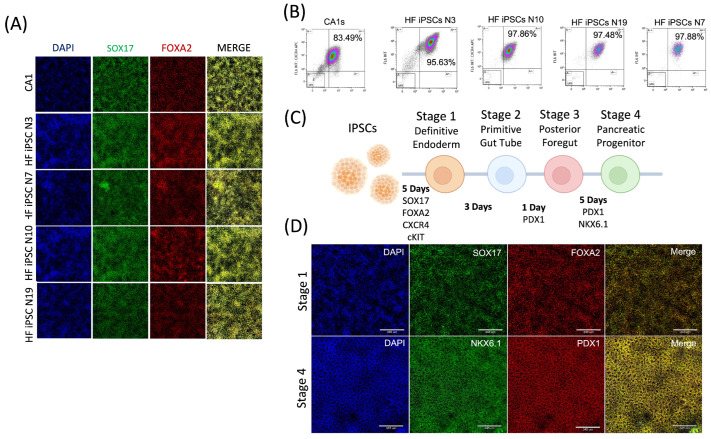
Induced pluripotent stem cells derived from cryopreserved hair follicles can be efficiently differentiated into definitive endoderm cells and pancreatic progenitor cells. All four cell lines were differentiated towards definitive endoderm cells using a 5 day protocol. The cells were then analyzed using immunofluorescence and compared to an embryonic stem cell (CA1) control. All the cell lines expressed SOX17 (Green = FITC488) and FOXA2 (Red = Cy5647) indicative of definitive endoderm cells (**A**). The cells were further evaluated through flow cytometry. All cell lines showed 95% or more double-positive expression of cell surface markers cKIT (PE) and CXCR4 (APC) (10× magnification) (**B**). A modified protocol was devised to differentiate the definitive endoderm cells towards pancreatic progenitor cells (**C**). The modified protocol resulted in NKX6.1 (Green-FITC488)- and PDX1 (Red-CY5647)-positive cells, as visualized using immunofluorescence (Scale bars = 345 μm) (**D**).

After the iPSCs had been successfully validated to be pluripotent, we aimed to study the clinical application of the cells by directly differentiating them towards definitive endoderm cells. It has been found that the transplantation of progenitor cells in mouse models can revert the effects of drug-induced β cell destruction [30]. Therefore, we aimed to create the cells that could be used in both an ex vivo and in vivo model for understanding Type I diabetes. To create definitive endoderm cells, iPS cells were seeded on Cultrex-coated plates and grown until 70% confluency in MTesR1 media. The media was then switched to endoderm basal media supplemented with an MR + CJ cocktail. The cells were then switched to basal media supplemented with a CJ growth factor cocktail on day two, and were kept in the media for 4 days. On the sixth day of differentiation, the cells were assessed for double-positive expression of cKIT^+^ and CXCR4^+^ using FLOW cytometry, and double-positive expression of SOX17 and FOXA2 using immunofluorescence (Figure 6A). The cell lines showed double-positive expression between 95.63% and 97.88% (Figure 6B). The cells were also taken towards pancreatic progenitor cells in a single trial. The stage 3 incubation was reduced from three days to one day for the iPSCs, resulting in a fourteen-day protocol (Figure 6C). The cells showed positive expression for NKX6.1 and PDX1 after stage 4, indicative of pancreatic progenitor cells (Figure 6D).

## 4. Discussion

Overall, our study aimed to understand the feasibility of using cryopreserved, plucked follicles as a starting source for autologous iPS generation. It was established that cryopreserved, plucked hair follicles have multiple selective advantages over other cell sources traditionally used for cell replacement therapies. Hair follicles from the scalp region can be taken from any individual at any age without the need for a medical professional, hospital infrastructure, or complex pieces of equipment.

The plucked hair follicle remains viable for extended periods of time in transport and cryopreservation. The immunophenotype of the follicle is also found to be consistent after cryopreservation. This was seen in the ability of patient samples to outgrow post-thaw from a wide array of individuals. The act of cryopreservation allows us to pull patient samples at the time of need or in advance of the treatment. It also allows us to arrest cells in the state at which they were frozen, decreasing the chances of those cells experiencing negative effects seen because of cellular aging [41]. The downside of cryopreservation in autologous manufacturing is the operational costs of running a high-quality biobank, but this may be outweighed by the benefit of having ‘younger’ cells obtained non-invasively and utilized in a timeframe that is efficient and effective. Cells may also be removed from a biobank and used to generate an autologous iPS biobank when companies have developed the tools, quality measures, and policies needed to ensure a bench-to-bedside experience.

Our optimized explant culture method allows for the selective expansion of keratinocytes from one plucked hair follicle. Across multiple participants, it was observed that one out of ten follicles were sufficient for the generation of millions of primary keratinocytes. This was consistent when the methodology was changed to using xeno-free, cGMP-compliant alternatives. Hair follicular cells confer multiple advantages when it comes to reprogramming. Previously in the literature, the cited drawback of using keratinocytes was the limited proliferation capacity in culture, which led to premature senescence [42]. However, our optimized protocol using DermaCult Keratinocyte Expansion media and rat-tail-collagen-coated plates led to multiple rounds of K14^+^K5^+^ cell expansion. It would be beneficial to perform lineage-tracing experiments from the primary outgrowth stage to see if the modified explant culture method selects for more proliferative, primary colonies.

Our reprogramming protocol led to the formation of at least one iPS colony for all participants by day 21. This mirrors the high transfection efficiency seen for keratinocytes when compared to other cell sources, such as fibroblast and umbilical cord tissue [43,44]. D Racila et al. showed that a transient expression of OCT4 was sufficient to differentiate keratinocytes towards neuronal and mesenchymal cell types, showing an increase in endogenous embryonic gene expression [45]. This, in conjunction with the understanding that keratinocytes 1. express much higher levels of endogenous c-Myc and KLF4 than fibroblasts, 2. do not need to go through the initial epithelial–mesenchymal stem cell transition, and 3. share more genes hypermethylated at CpGs with human embryonic stem cells (ESCs) than other somatic cells, indicates that keratinocytes may be able to convert into iPS cells much faster than other cell sources [36,46,47]. Additionally, keratinocytes from plucked hair follicles, as opposed to skin biopsies, are not directly impacted by prolonged UV damage, which impacts the functionality of the living cells. In the skin, prolonged UV damage leads to collagen fiber damage and the accumulation of mutation in the cell’s DNA [48]. The hair follicle is different from the skin cell, as it contains layers of protection to prevent direct UV impact. Finally, we observed that keratinocytes, when placed in calcium rich, reprogramming media, start to terminally differentiate and do not persist in culture, leaving only reprogrammed cells to proliferate. This reduces the chance of human error involved in picking colonies. These selective advantages, combined with future technologies and optimized cell culture protocols, can decrease the manufacturing burdens experienced in autologous iPS generation. To improve the chances of having more than one colony formed for each participant for autologous manufacturing, it may be beneficial to test the addition of ascorbic acid, which has been linked to higher reprogramming efficiencies in umbilical cord tissue reprogramming [25]. Mohamed et al. found an eight-fold increase in the number of colonies when cells were treated with ascorbic acid prior to reprogramming [25].

Simultaneously, our study considers the impact of donor age and sex throughout the process of generating iPSCs. From our studies, it was hinted that cellular outgrowth from plucked hair follicles had no significant correlation with age, and outgrowths were still observed for our oldest participant, who was 77 years of age. This indicates that older individuals can still have their hair follicles collected and banked on the chance that they can be used for regenerative medicine treatments soon. There was, however, a difference in outgrowth success when comparing males and females. The decline in outgrowth success for older women could be a result of hormonal changes because of menopause. The hair cycle and hair follicle structure are highly affected by various hormones. Hormonal changes, as frequently observed at menopause, can lead to female pattern hair loss (FPHL) [49]. This causes a decrease in hair density and diameters. It is hypothesized that this decrease is due to the gradual decrease in androgen secretion and the decline in the levels of estradiol and progesterone after menopause [49]. A study comparing scalps of women aged 22 to 70 years of age found that older women’s hair follicles were, on average, more shrunken, and that this correlated with genetic mutations in the follicle stem cells [49]. A study conducted by William et al. found that there are multiple age-related changes in the aging female scalp that can impact hair growth [50]. The group observed that these changes were observed in the fibroblast population of cells, the dermal papilla (DP), and the dermal sheath (DS) [50]. It is unknown whether the keratinocyte population in the follicle is equally affected. To further elaborate on these findings, it would be beneficial to do a larger scale experiment with women pre- and post-menopause and evaluate their outgrowth rate using our protocol. Another beneficial study would be to look at women in the older cohort who are currently on hormone replacement therapy (HRT) and test their outgrowth rates against women who are not on HRT.

Donor age did not have an impact on our ability to generate stable iPSCs. IPS cell lines from individuals over the age of 60 and younger than 40 both showed consistent expansion in culture and remained stable, as evinced from the karyotyping analysis. All of the cell lines also directly differentiated towards definitive endoderm cells, with no major qualitative differences. Our results are reflective of the literature findings which suggest that donor age may reduce reprogramming efficiency but has not been found to affect iPSC maintenance or differentiation capacity, with the possible exception of the accumulation of mutations in mitochondrial and nuclear DNA [51]. The current body of evidence regarding the impact of donor age on iPSC differentiation capacity is limited by the small number of iPSC lines studied, which results in statistical and methodological limitations. Therefore, comprehensive and systematic studies are required to assess the effect of donor age on iPSC differentiation capacity using a larger number of iPSC lines produced and maintained under uniform conditions to yield a conclusive answer.

Another finding from this study came from the data set wherein cellular outgrowth was compared to reprogramming efficiency. There was a strong positive correlation between outgrowth rate and colony-forming units. Since the starting number of cells was kept constant, there is an implication that certain cells have a selective advantage for reprogramming. This cell population may also be driving cellular outgrowth. In the work conducted by Shakiba et al., it was found that in heterogeneous populations of MEFs, cells derived from Wnt1-expressing cells, representative of a neural crest population, gave rise to dominant clones during reprogramming [52]. The authors concluded that the reprogramming “elitness” exhibited by these cells was linked to their ability to reprogram more efficiently, allowing them to transition to an iPS state more rapidly.

The regenerative medicine potential of our cells comes from their ability to be used as a robust starting material. We have shown that the plucked hair follicle can be collected and cryopreserved. The cryopreserved follicles can then be used to generate iPS cell lines from multiple participants. These stable cell lines can be directly differentiated towards the endoderm lineage. Specifically, they can be used to create definitive endoderm cells, which have the potential to be differentiated into NKX6.1+/PDX1+ cells in vitro. When optimizing the protocol for definitive endoderm differentiation, our lab found that HF-iPS cells require a greater timeline for complete differentiation than previously cited in [34,35]. The cells were kept in the definitive endoderm basal media for 5 days to achieve greater double-positive expressions of cKIT and CXCR4. Overall, our optimized protocol allows us to generate sufficient quantities of progenitor cells for ex vivo and in vivo applications. For example, patient-specific pancreatic progenitor cells can be transplanted in streptozotocin (STZ)-treated diabetic mice to recover insulin deficiency [53]. Any regeneration of beta cells or insulin production post-transplantation would be a direct result of pancreatic progenitor cell differentiation. This can be used to create in-human studies for the treatment of Type I diabetes in the future.

## 5. Conclusions

As the field of regenerative medicine rapidly advances, we are closer to regrowing, repairing, and replacing damaged or diseased cells and, in turn, restoring the body to its normal functionality. The key to unlocking this potential lies in our own cells, which deliver the greatest potential with the highest safety margins when it comes to patient care. Our own cells act as the starting material for screening drug candidates, autologous cell replacement therapies, as well as organ generation. In the past decade, the hair follicle has unquestionably emerged as a viable, diverse tissue source with exceptional potential. Its accessible nature, efficient collection procedures, and versatile stem cells underscore its unique position in the realm of regenerative applications. With the incorporation of specialized culture media and cryopreservation techniques, hair follicle-derived cells can be harnessed by researchers and individuals worldwide, amplifying the scope of exploration and eventual therapeutic interventions.

## Data Availability

All data are contained within the manuscript and Supplemental Information Section.

## Supplementary Materials

The following supporting information can be downloaded at: https://www.mdpi.com/article/10.3390/cells13121010/s1, Table S1: Participant Demographic; Table S2: Conjugated Probes for Flow Cytometry; Table S3: Fluorescent Conjugated Probes for Immunofluorescence.
