# Supplementary material for: Efficient Generation of Pancreatic Progenitor Cells from Induced Pluripotent Stem Cells Derived from a Non-Invasive and Accessible Tissue Source—The Plucked Hair Follicle
Source: Cells. 2024 Jun 10;13(12):1010. doi: 10.3390/cells13121010 (PMC11202038; doi:10.3390/cells13121010)
Supplement: Supplementary file 1 [file cells-13-01010-s001.zip › cells-2998728-supplementary.pdf]

*Supplementary Table S1: Participant Demographics*

| N | Sex | Age | N | Sex | Age |
|---|---|---|---|---|---|
| **1** | Female | 26 | **15** | Male | 32 |
| **2** | Male | 27 | **16** | Female | 57 |
| **3** | Male | 28 | **17** | Female | 48 |
| **4** | Female | 59 | **18** | Male | 43 |
| **5** | Female | 23 | **19** | Female | 28 |
| **6** | Female | 30 | **20** | Male | 42 |
| **7** | Male | 64 | **21** | Female | 27 |
| **8** | Male | 38 | **22** | Female | 44 |
| **9** | Female | 58 | **23** | Male | 34 |
| **10** | Female | 66 | **24** | Male | 34 |
| **11** | Male | 77 | **25** | Female | 31 |
| **12** | Male | 48 | **26** | Female | 26 |
| **13** | Male | 34 | **27** | Male | 36 |
| **14** | Male | 30 | **28** | Female | 27 |

*Supplementary Table S2: Conjugated probes for FLOW cytometry*

| Antibody | Company Name, Catalog #, Lot # | Conjugation | uL/ 100 uL sample |
|---|---|---|---|
| CXCR4 | BioLegend, Clone 12G5, Catalog #306510, Lot #B359425, USA | APC | 5 |
| cKit | BioLegend, Clone S18022G, Catalog #375206, Lot #B360191, USA | PE | 20 |
| CXCR4 | Stem Cell Technologies, Clone 12G5, PE, Catalog #60089PE.1, Lot #BX29941, USA | PE | 5 |
| cKit | BioLegend, Clone 104D2, Catalog #313205, Lot #B271240, USA | APC | 5 |
| CD90 | Invitrogen, Clone eBio5E10, Catalog #25-0909-41, Lot #2459731, USA | PE-C7 | 5 |
| CD105 | BioLegend, Clone 43A3, Catalog #323208, Lot #B315549, USA | APC | 5 |
| CD73 | BioLegend, Clone AD2, Catalog #344004, Lot #B248075, USA | PE | 5 |
| CD44 | Invitrogen, Clone IM7, Catalog #11-0441-82, Lot #2208880, USA | FITC | 5 |

*Supplementary Table S3: Fluorescent conjugated probes for immunofluorescence*

| Primary Antibody | Company, Catalog #, Lot # | Dilution | Secondary Antibody | Dilution |
|---|---|---|---|---|
| **K14 Polyclonal** | Invitrogen, Catalog #PA5-16722, Lot #VJ3109866, USA | 1:100 | Alexa Fluor 647 goat anti-Rabbit IgG (H+L) | 1:1000 |
| **K5 Monoclonal** | Invitrogen, Clone XM26, Catalog #MA5-12596, Lot #XE3589264, USA | 1:100 | Alexa Fluor 488 goat anti-Mouse IgG (H+L) | 1:1000 |
| **OCT4 Polyclonal** | Invitrogen, Catalog #PA5-27438. Lot #XC3518081A, USA | 1:100 | Alexa Fluor 488 goat anti-Mouse IgG (H+L) | 1:1000 |
| **NANOG Polyclonal** | Invitrogen, Catalog #PA1-097, Lot #WH333784, USA | 1:100 | Alexa Fluor 488 goat anti-Rabbit IgG (H+L) | 1:1000 |
| **SOX2** | R&D Systems, Clone 245610, Catalog #MAB2018, Lot #KGQ0319021, USA | 1:100 | Alexa Fluor 647 goat anti-Mouse IgG (H+L) | 1:1000 |
| **SOX17 Monoclonal** | Invitrogen, Catalog #MA5-24891, Lot #XJ3729211, USA | 1:100 | Alexa Fluor 488 goat anti-Mouse IgG (H+L) | 1:1000 |
| **FOX2A Monoclonal** | Invitrogen, Catalog #701698, Lot #2545822, USA | 1:100 | Alexa Fluor 647 goat anti-Rabbit IgG (H+L) | 1:1000 |
| **ALPHA-SMA Monoclonal** | Invitrogen, Catalog #MA5-41117, Lot #YB3840615B, USA | 1:200 | Alexa Fluor 488 goat anti-Rabbit IgG (H+L) | 1:1000 |
| **Anti-Hu Brachyury** | Invitrogen, Clone X1A02 Catalog #14-9770-82, Lot #2469564, USA | 1:100 | Alexa Fluor 647 goat anti-Mouse IgG (H+L) | 1:1000 |
| **TUJ-III** | EMD Millipore, Clone TU-20, Lot #3834818, USA | 1:100 | Alexa Fluor 488 goat anti-Mouse IgG (H+L) | 1:1000 |
| **PDX1** | Abcam, Catalog #ab47308, Lot #GR3437451-1, USA | 1:200 | Goat anti-guinea pig IgG 594 | 1:1000 |
| **Anti-hNKX6.1 Monoclonal** | R&D Systems, Clone 631438, Catalog #MAB5857, Lot #CEFK0222011, USA | 1:100 | Alexa Fluor 488 goat anti-Mouse IgG (H+L) | 1:1000 |
